# Clinicians’ perspectives on integrating smartphone application data into routine alcohol dependency treatment: factors influencing implementation

**DOI:** 10.1186/s13722-025-00597-4

**Published:** 2025-08-13

**Authors:** Josefine Östh, Andreas Lundin, Peter Wennberg, Sven Andréasson, Anna-Karin Danielsson

**Affiliations:** 1 Department of Global Public Health, Karolinska Institutet, Stockholm, Sweden; 2 Centre for Epidemiology and Community Health, Region Stockholm, Stockholm, Sweden; 3 Department of Public Health Sciences, Stockholm University, Stockholm, Sweden; 4 Department of Psychology, Inland Norway University of Applied Sciences, Lillehammer, Norway; 5 Centre for Psychiatry Research, Department of Clinical Neuroscience, Karolinska Institutet, Solna, Sweden

**Keywords:** Alcohol treatment, Mobile health, Smartphone, Attitude of health personnel, Focus group discussion

## Abstract

**Background:**

Incorporating clinicians’ perspectives is essential for the successful implementation of novel interventions in health care. This study aimed to explore clinicians’ experiences of using smartphone-derived data in alcohol dependency treatment, and factors affecting implementation into routine care.

**Methods:**

Two focus group discussions were conducted in April 2023, including 10 clinicians working at a specialist addiction clinic in Stockholm, Sweden. The clinicians had various levels of experience using smartphone-based data, which was available through two online portals, as part of a randomised controlled trial evaluating two smartphone-based interventions. Data were analysed using Thematic Framework Analysis, guided by Normalisation Process Theory.

**Results:**

Two main themes were identified: *The patient as the driving force* and *Cultivating commitment*,* competence and credibility*. The first theme highlighted a person-centred approach that permeated the practice and how the patients’ engagement with the app-based interventions guided the clinicians’ own involvement. Benefits of the interventions for both patients (i.e., increased awareness and control) and clinicians (i.e. supportive during treatment sessions) were also acknowledged. Clinicians believed that the interventions offered an opportunity for patients to become more actively involved in treatment and noted that clinician access to the app-derived data was less important. The second theme covered clinician discussions on the need for support and guidance to make better use of the interventions, continuity in the work, and additional work time. Moreover, the use of external portals made the intervention less accessible. Potential risks and concerns with the interventions were raised, including technical instability and data security.

**Discussion and conclusions:**

The results of this study indicate that a breathalyser-coupled and a drink-counting smartphone application have potential to be supportive complements to alcohol dependency treatment. According to the clinicians, the app-based interventions enhanced patient accountability in the change process and supported treatment delivery. To be effectively implemented into routine care, using a person-centred approach is key, as well as ensuring optimal conditions for clinicians to effectively use the systems. Technical issues constitute a barrier to acceptance, why technical robustness must be ensured.

**Supplementary Information:**

The online version contains supplementary material available at 10.1186/s13722-025-00597-4.

## Background

Mobile health (mHealth) refers to health care supported by mobile devices and has become a central component of health care delivery in the past years [1]. The pivotal role of digital interventions in health system strengthening has been acknowledged by the World Health Organization (WHO), demonstrating potential benefits of mHealth in increasing access to, and quality of, health care services [2, 3]. Smartphone applications (apps) are included in the concept of mHealth and may be especially useful given the extensive global use of smartphones [2]. Furthermore, apps have specific attributes (e.g., timely reminders, prompt self-monitoring, visualisation of progress and goal, linking to external devices) that are suitable for providing tailored behaviour change interventions [4–6]. Within a treatment setting, such as for example for alcohol dependence, using apps could also be beneficial to overcome stigma, increase treatment engagement and adherence, and facilitate communication with health care providers [5, 7, 8].

In recent years, several apps have been developed to reduce the large alcohol-related burden. Alcohol use is estimated to cause 2.6 million global annual deaths [9] and is fully or partially associated with over 200 different diseases or injuries, including alcohol dependence [10]. Previous evidence on the effectiveness of apps to curb alcohol consumption is, however, inconsistent, and firm conclusions are limited by the use of different apps with different content and features, intended for different populations [11]. Yet, recent studies show non-trivial consumption reducing effects in both hazardous [12–14] and alcohol-dependent [15–17] populations. According to a recent meta-analysis, offering digital and remote interventions for substance use disorders have promising effects, especially when provided as a treatment supplement [18]. Similarly, app-based interventions provided as adjuncts to alcohol treatment have a small additive effect compared to standard treatment only, although the need for additional comprehensive studies with higher quality has been emphasised [7].

In a recent randomised controlled trial (RCT) [15], we tested the effectiveness of a drink-counting and a breathalyser-coupled app as adjuncts to standard treatment for alcohol dependence. Both apps were connected to online portals where data could be reviewed by clinicians and was used for feedback in discussions with patients during treatment sessions. Results showed that those randomised to use the breathalyser reduced their heavy drinking to a larger extent than those randomised to standard treatment only [15]. The usefulness of both apps was further highlighted in patient interviews, although usage was partly reduced by primarily technical barriers [19].

While considering the potential of apps in reinforcing health care, there is need for evidence regarding usefulness in clinical practices. Relatedly, successful implementation and continuity of mHealth interventions are highly dependent on health care clinicians [20]. Studies on clinician perceptions are therefore necessary to improve the understanding of factors impacting the implementation and feasibility of such interventions, while also considering the contexts in which they are tested [3].

Prior studies have proposed a conceptual framework including factors affecting clinicians’ adoption of mHealth interventions (i.e., engagement, training, infrastructure, costs, motivation, system utility) that needs to be considered prior to implementation [21]. Moreover, a supportive organisation, effective leadership, and clinician skills have been highlighted as critical factors within the digital transformation of health care [20].

Looking specifically at app-based interventions targeted at excessive alcohol use or dependence, previous studies show that patients in both primary care and addiction aftercare desire apps that are integrated in treatment, with staff invested in their app use, and apps being part of the treatment plan [22, 23]. Results further show several health and cost benefits of scaling up app-based alcohol interventions, especially within primary care [24]. According to clinicians, the most common barriers to adopting app-based interventions targeted for patients with problematic substance use or substance use disorders in primary care seem to be limited time, shortness of staff, and intervention complexity [22, 25, 26]. Studies on clinician views on app-based interventions within specialised addiction care are lacking. However, a recently published study explored clinicians’ views on standalone internet-delivered interventions [27]. While the results emphasised that such interventions can reduce the large treatment gap for alcohol dependence and provide more flexible and tailored care, the need for structured workflows and communication between different care units (e.g., digital and in-person contacts), was underlined [27].

To the best of our knowledge, there is lack of studies exploring clinician’s perspectives of integrating different app-based interventions for use within specialised alcohol addiction care, which motivates further investigation.

In this context, Normalisation Process Theory (NPT) offers a valuable framework for gaining deeper insight into how such interventions can become embedded or “normalised” into routine clinical practice [28]. By focusing on the actions of clinicians, both individually and collectively, NPT helps identify potential barriers to successful implementation, making it particularly useful for studying the integration of novel health care interventions [28].

The aim of this study was two-fold. First, we aimed to explore clinicians’ experiences of using smartphone-derived data in the treatment of alcohol-dependent patients. Second, by applying an adaptation of NPT, we aimed to examine factors influencing implementation of mHealth into routine care.

## Methods

### Study design

A qualitative focus group discussion (FGD) design was chosen. This approach was considered central in elucidating clinicians’ experiences, allowing them to express, explore, and expand on their ideas in discussions with each other [29].

### Theoretical framework

NPT was chosen as theoretical perspective guiding the analysis and interpretation, as it focuses on the implementation of complex interventions [28]. It includes four main components, or constructs (*Coherence*, *Cognitive participation*, *Collective action*, *Reflexive monitoring*) (Table 1), that can be applied during either development, evaluation, or implementation of an intervention [28]. NPT has been applied in various studies on the implementation of health care interventions, guiding the understanding of critical factors for success or failure [30].

**Table 1:** Normalisation process theory (NPT) constructs and pre-defined codes, and open codes, with descriptions

Construct^a^	Description^a^	Pre-defined codes^b^	Code description^b^	Exemplary quote
Coherence	Understanding and sense-making of intervention in practice	Differentiation	How clinicians think the intervention distinguishes from their standard way of working	*" It’s difficult to just dive in and mix your old methods with a completely new one"*
		Communal specification*	How clinicians agree on the purpose of the intervention	*N/A*
		Individual specification	How individual clinicians understand what the intervention requires from them	*“It was new to me working with people that did not want complete abstinence"*
		Internalisation	How clinicians perceive the value of the intervention	*“It is a great starting point for discussion"*
Cognitive participation	Commitment and engagement to intervention	Initiation	How key clinicians drive the intervention forward (role of leaders etc.)	*“It got better when you [study coordinator] took over so we did not have to keep in contact with them [app developers]”*
		Enrolment	How clinicians join in with the intervention	*“I must see what I can get out of it…or be a believer*,* ‘this app will give me what I need in my work’"*
		Legitimation*	How clinicians agree the intervention is the right way forward and should be part of their work	*N/A*
		Activation	How clinicians continue to support the intervention (suggestions for moving forward)	*“I think that if you are going to work with a tool like this*,* you should use it every day and perhaps with most patients*,* so you become familiar with it"*
Collective action	Working together to make the intervention function	Interactional workability	How clinicians do the work required by the intervention	*“Instead of incorporating it into my way of working I chose to treat it as something the patients handled on their own"*
		Relational integration*	How the use of the intervention affects the confidence clinicians have in each other	*N/A*
		Skill-set workability	How the work is allocated to clinicians (training, support and education needed)	*“I had big troubles logging in to the portal*,* so I could not access the data"*
		Contextual integration	How the intervention is supported by the clinic’s organisation (organisational support)	*”I needed extra time to work with it [portal]*,* before I met the patient”*
Reflexive monitoring	Reflections on or judgement of intervention	Systematisation*	How clinicians retrieve information/feedback on the effects of the intervention	*N/A*
		Communal appraisal*	How clinicians collectively value the intervention as worthwhile	*N/A*
		Individual appraisal	How individual clinicians value the intervention as worthwhile	*“If we are going to use this*,* it should function perfectly*,* because then they [the patients] will trust it"*
		Reconfiguration	How clinicians adapt their work following the perceived value of the intervention	*“I usually inform all new patients who start treatment about Glasklart [drink-counting app]*,* for registering"*
		**Open code**	**Open code description**	**Exemplary quote**
		Adaptations	How the intervention is and needs to be adapted following patient engagement, goals, and needs	*“It should be guided by the patient*,* by what the patient thinks is good"*
		App limitations	How malfunctions or lack of certain features affect the intervention	*“I had to spend many conversations discussing how poorly it [the breathalyser] worked*,* and it was going to be sent back*,* and they’d get a new one*,* like these problems sometimes took over"*
		Patient benefits	How clinicians describe the intervention as beneficial for the patients	*“She blew*,* and she knew*,* ‘at four o’clock I’m going to blow’. Those were her critical hours*,* and just knowing that she wanted to blow zero*,* made her abstain"*
		Patient responsibilities	How the intervention could help activate patients in treatment, clinician access to data less important	*“It gets pretty clear that you’re supposed to work with something in between the [treatment] sessions"*
		Potential harms	How clinicians discuss intervention-related harms	*“And then he understood that he could trick the system*,* and so*,* it [alcohol * *consumption] didn’t correspond too well"*

a = Murray et al. (2010) [28]; b = May et al. (2022) [34]; *=pre-defined code not applicable

### Participants and setting

Clinicians working at a specialist clinic (Riddargatan 1) within the Stockholm Centre for Dependency Disorders were invited to participate. Eligibility criteria were having experience of working with patients that were randomised to either one or both apps used in the clinical trial that was ongoing between September 2020 and July 2023. The clinic has previously been described in detail [15, 19].

In total, 16 clinicians were introduced in both app-based systems during this period, including registered nurses, nurses specialised in psychiatric care, licenced psychologists, and social workers. Eleven clinicians accepted participation, two declined because of time constraints, and three were not asked as they had terminated their employment at the clinic over a year before (*n* = 2) or had not had any study patients in treatment despite being introduced (*n* = 1).

Clinicians were divided into two groups based on their availability, with five and six clinicians in each group. One clinician later had to cancel participation due to unforeseen events, leaving five in each group. The sample comprised six women and four men, with a median age of 51 years (range 40–68). Four were psychologists, four were registered nurses, and two were social workers (Table 2). All clinicians were given access to both app portals. However, their experience using the systems varied: some had many patients in treatment using the apps, others had only a few, and two clinicians had experience with only one of the two app-based interventions.

**Table 2:** Characteristics of the clinicians participating in the focus group discussions

Clinician #	Focus group	Profession
1	1	Psychologist
2	1	Registered Nurse
3	1	Registered Nurse
4	1	Social worker
5	1	Registered Nurse
6	2	Psychologist
7	2	Social worker
8	2	Registered Nurse
9	2	Psychologist
10	2	Psychologist

### App-based interventions

The 12-week intervention was ongoing from September 2020 to June 2023 and has previously been described in detail elsewhere [15, 31]. In brief, patients were allocated to treatment as usual (TAU), TAU + a drink-counting app, or TAU + a mini breathalyser connected by Bluetooth to an app used for receiving prompts and taking breath alcohol concentration (BrAC) tests. The sample included 162 alcohol dependent patients, with moderate levels of dependence severity and no major psychosocial problems or severe somatic diseases. The majority had never received treatment for alcohol problems before signing up for the study. Clinicians could access and review the patients’ app-data in two separate online portals, one comprising daily self-registered alcohol use over a three-month period, and the other BrAC tests taken trice daily over the same period. They were instructed to review their study patients’ data in the portals at least in conjunction with each individual treatment session, for use as a basis for discussion. Hence, they were not requested to regularly check the data between treatment sessions. See Supplement 4 for an example of app and portal interfaces.

### Data collection

Data were collected at the clinic in April 2023. The first author moderated the discussions, and a research assistant was the facilitator, taking notes on non-verbal communication. All clinicians, including the moderator and secretary, were seated in a circle to facilitate the discussion [32]. Topics for discussion were pre-specified in the interview guide (Supplement 1) and included how the app-derived data were used, their pros and cons, whether the apps were perceived as helpful for the patients, and the perceived value of using digital tools in treatment. The interview guide was used flexibly during the audio-recorded FGD’s, that lasted for 67 and 63 min, respectively. No compensation for participation was provided, apart from coffee and snacks.

### Data analysis

Data were analysed using Thematic Framework Analysis, as described by Gale et al. [33]. Considering that our aim included both to explore clinicians’ views and to identify critical factors for implementation by using the NPT, we applied a combination of inductive and deductive approaches in our analysis.

First, data were transcribed by an external research assistant and the first author. To familiarise with the data, the transcripts were read and re-read separately by the first and last author. Audio files were revisited and field notes on non-verbal communication were read through. Key ideas and impressions emerging from this process were also documented.

When the two researchers had gained sufficient familiarity with the data, they met to discuss their first impressions and initiated the coding process. Primarily, the NPT code manual as suggested by May et al. [34] was used, and complemented with open coding by the first author, to ensure that no important perspectives got lost [33]. Table 1 provides an overview of the constructs and pre-defined codes included in the NPT, and the codes derived from open coding. All pre-defined codes were not applicable (Table 1).

Followingly, the two researchers agreed on a final set of codes (each with brief explanations as suggested by Gale et al. [33]. Codes were grouped into categories by the first author, with detailed descriptions. Data were then charted into a matrix that was used for interpreting the data [33] (Supplement 2 for an example). Although interviews and transcripts were performed in Swedish, the coding process and subsequent analysis were conducted in English. Quotations were translated from Swedish to English. The translations were refined after discussion with a native English-speaking colleague, also fluent in Swedish.

Themes were generated inductively by discussions in relation to the matrix content, while also considering initial ideas and reflections, and the aim of the study. NPT was used to deductively map and frame the findings. All authors gave feedback on the categories and themes that derived from this process. Together, the authors represented a multi-professional team with broad academic/clinical expertise on addiction and different professional backgrounds (i.e., in nursing, medicine, psychology, and public health) (Supplement 3 for detailed description [35–37]).

## Results

Two main themes were generated: (1) *The patient as the driving force*, including two categories (*Balancing different patient needs* and *Active and empowered patients*), and (2) *Cultivating commitment*,* competence and credibility*, including two categories (*Optimising clinician opportunities* and *Trusting the system*).

### Theme 1: the patient as the driving force

Applying a person-centred approach was the norm at the clinic, and the clinician-patient alliance was highly valued. Therefore, clinicians adapted the treatment to meet each patient in their motivation, why each patient’s engagement with the apps determined the degree of clinician involvement. The clinicians considered the use of apps valuable for both patients and themselves and described several profitable features of the app-based interventions. The use of apps further offered an opportunity for patients to stay active in between treatment sessions, which was considered a strong rationale for using the interventions. It was suggested by some clinicians that the apps could be used without their involvement, unless specifically requested by the patient, to further stress that the patient is responsible for making the desired change.

#### Category 1 A: balancing different patient needs

Clinicians described how all patients have different needs and thus require different treatment, a central approach in their work as therapists. Thus, there was a need to tailor treatment for each patient to meet the patient in their own motivation and engagement. Relatedly, it was the patients’ engagement with the interventions that decided the level of use during treatment sessions.

”It was absolutely the patients’ engagement with the app that determined whether it was more or less [discussed]” (Psychologist, focus group 2).

Some incorporated the interventions into their practices and even advised other patients to use a drink-counting app or a breathalyser, as it was considered helpful. However, some considered the interventions less valuable or even burdensome for the patient and believed it did not align with the patient’s interest, although patients had signed up themselves to partake in the study. Others considered the app-based interventions challenging to integrate into treatment. This often led to simply asking the patients if the drink-counting app or breathalyser was used or not. Nonetheless, asking the patients *how* the apps were used was believed to be essential to engage and motivate the patients. Asking the patients what would best support them in their work toward behaviour change was underlined as a factor for success, as was the importance of working actively with the app or breathalyser during treatment sessions, for example by connecting the interventions to both internal and external motivation.

“So, it’s not just really about showing the results, but constantly trying to refer back to ‘right, so you slept better during the night this week’?” (Psychologist, focus group 2).

#### Category 1B: active and empowered patients

Clinicians identified various beneficial aspects of the interventions. For example, the apps facilitated continuous registrations, patient self-analysis of consumption, tracking of days with zero consumption, and increased patient control (to reduce or abstain). Some clinicians assumed that the apps’ effectiveness was most prominent in the first few weeks, and that patient motivation to use the tools then declined.

“There are many [patients] that use the [drink-counting] app as a moderation strategy. You buy yourself time, postponing the decision to drink. Like ‘I’ve had 3 drinks tonight, should I really have a fourth?’ That’s been excellent” (Registered Nurse, focus group 1).

It was believed that patients made greater efforts to register their consumption using the apps, compared with the paper calendar. Relatedly, clinicians experienced that patients who used the apps to track their consumption were more aware of their consumption patterns. Thus, treatment sessions could move more quickly to discussions on, for example, risky situations and setbacks.

They further discussed how apps could be profitable in general, for example by the extended reach to the population at large. While the drink-counting app mainly was considered a simple and convenient way to register consumption, clinicians perceived that the breathalyser evoked a certain interest and curiosity among the patients, and it was described as having great potential.

The portals were generally considered informative and supportive, as well as motivating for their patients, and a valuable addition to routine care. Technical problems experienced by patients were also seen as alliance-building as it enabled clinicians to meet and validate the patient’s frustration.

“I think it’s been pretty good, this portal, when they’ve blown [used the breathalyser] and to be able to log in and see these curves and charts. It motivated one of my patients a lot, and for the other one who had a goal of moderate consumption, also quite motivating. Then you could roughly see how much they drank, she’s at this blood alcohol level approximately, and then you could follow that in relation to her goals and that’s also quite good” (Psychologist, focus group 2).

Still, clinicians discussed for whom the information in the digital systems was intended and underscored the patient’s responsibility for making the desired change. The apps were perceived helpful in clarifying this responsibility, as well as the work to be done in between treatment sessions.

”I feel that some patients, even though we usually give them home assignments and they have material to work with, they’re a little inactive between sessions. They see the therapy session as the active part, after which they relax, like after a workout. But that’s not how we see it. We think it should be part of daily life and here [by using the app], it becomes a very tangible thing, that it enters everyday life” (Social worker, focus group 2).

For some clinicians, having access to the patients’ data was considered inappropriate, although patients did not seem to mind. Contrary, others thought that patients might appreciate the increased control introduced by the interventions. The prospect of involving relatives in receiving the information, for those patients that want this type of control, was discussed. Clinician access to the information was by some thought less important.

”What’s important is that they’ll get the result […]. And it’s that approach I think we need, that it’s the patient’s responsibility. They can write it down and tell us. If they write it down wrongly, it’s still an intervention” (Social worker, focus group 1).

The approach of letting the patient be in charge of the apps was described as a method for future patient interactions at this clinic, developing a more patient-centred image of addiction care.

”Most of us who have been [working] at other addiction clinics […], then you’re used to it being for the sake of the clinician. ‘I use a breathalyser so that *you* can write down the results’. That’s the first thing you do, you use a breathalyser before group treatment, before receiving meds, before giving samples. But here [at this clinic], you do it for *your own* sake and I won’t actually check this” (Registered Nurse, focus group 1).

### Theme 2: cultivating commitment, competence and credibility

Delivery of the interventions was hindered by clinicians’ pre-conceptions, and patient’s negative experiences as perceived by the clinicians, as well as time constraints and inaccessibility. Continuity in working with new methods was described as essential for embracing the interventions, as was the need for support and simplicity. Technical problems with particularly one of the interventions were believed to affect trustworthiness and taking time from treatment sessions with patients. Additionally, potential risks were identified and discussed.

#### Category 2 A: optimising clinician opportunities

Engagement with the interventions was influenced by pre-conceptions on what kind of intervention that would suit their patients, as well as patients’ experiences of technical problems, and problems signing in to the portals. There was a need to value the interventions as beneficial, for the patient, but also for each clinician working with them, to get them onboard.

“So, if it’s [the portal] going to be a relevant add-on, it perhaps needs to have a real function for me as a clinician. *I* must see what I can get out of it…or be a believer, ‘this app will give *me* what I need in my work’” (Psychologist, focus group 2).

Clinicians underlined the importance of having a dedicated person (i.e., the study coordinator) supporting them in the implementation of the interventions. Relatedly, clinicians described that they were not prepared to be a “technical helpdesk” to solve various issues experienced by patients. Some also discussed their lack of deeper knowledge as a barrier for engagement, and desired support on how to work flexibly with the interventions in relation to patients with various interest and needs, as well as simpler, more accessible interventions. Relatedly, reviewing the data together during digital treatment sessions was suboptimal, as the shared screen was too small. Clinicians further discussed the need to work more intensively and continuously with the interventions, and some suggested the introduction of designated app-therapists (i.e., focussing on/being knowledgeable of a specific system) as a possible solution.

”I think that if you are going to work with a tool like this, you should use it every day and perhaps with most patients, so you become familiar with it” (Psychologist, focus group 2).

Furthermore, some clinicians experienced that the interventions required extra time that was not compatible with the routines at the clinic, with many patients and shortages of time.

”I needed extra time to work with it [portal], before I met the patient” (Registered Nurse, focus group 1).

#### Category 2B: trusting the system

This category covered the clinicians’ views on the interventions as being worthwhile. Here, they identified several areas for improvement, and factors that might affect the expected added value of the interventions.

For example, although it was possible to re-direct the camera when taking BrAC tests, many patients did not, and clinicians considered this photo offensive and counterproductive (i.e., seeing negative pictures of yourself). Relatedly, the use of prompts (drink-counting app and breathalyser) was identified as a potential risk of negative consequences (i.e., makes you think about alcohol). Furthermore, security concerns of app-data stored in cloud services were expressed, leading to an additional responsibility to safeguard this data. However, there was a general belief that this did not seem to distress the patients.

Importantly, the breathalyser app-based intervention was considered technically unstable and unreliable by some of the patients, and consequently also by the clinicians. Clinicians described how unreliable results affected their patients’ motivation negatively, and how discussions on technical problems took valuable time from treatment sessions.

“I had to spend many conversations discussing how poorly it [breathalyser] worked, and it was going to be sent back, and they’d get a new one, like these problems sometimes took over. Yes, but okay, there was so much to discuss about these issues” (Registered Nurse, focus group 1).

Another identified risk with breathalyser data was tolerance. One clinician described a situation with a patient who had developed tolerance and hence did not feel intoxicated at a given level. This was by the patient interpreted as a cue to consume more, and made the clinician reflect on how we can detect patients who might not benefit from this type of interventions.

“It’s almost like then they think that ‘it wasn’t as bad as I thought it would be’, and I also think that tolerance as a concept is sometimes difficult for patients to grasp” (Psychologist, focus group 1).

Clinicians’ distrust in the BrAC output led to feelings of discomfort (i.e., difficult knowing whether to trust the output or the patient). False security was also brough forward as a potential risk within the treatment setting, where the output was considered reliable only if the breathalyser was used continuously and sincerely (e.g., risk of false sobriety, misleading external control). It was considered important to discuss consumption levels and not just trusting the output seen in the portal.

“I also had a great discussion with a patient that proved [sobriety], sent pictures and results to his wife. And then he understood that he could trick the system, and so, it [alcohol consumption] didn’t correspond too well. But it was nice, he was open with me about it, and we discussed ‘what is it that determines what you do and your sobriety, the external proof of the breathalyser, or to your wife, or do you want to change and like…?’” (Registered Nurse, focus group 2).

Surveillance of patient data in addiction care was further discussed in relation to routine work at the clinic, where it was considered as potentially harmful for the patient-clinician alliance, given the sensitivity of alcohol use.

“What I find central is ‘What’s the function?’ Is it external control? Generally, treatment and research aim to enhance the patient’s own motivation. At this clinic, the starting point is to always trust the patients based on what they say and feel comfortable conveying to us. Adding a breathalyser to this must be guided by patients’ need of support” (Psychologist, focus group 2).

Contrary, some clinicians clarified that a control system with objective data might be desired by some patients but that it necessitates a technically stable system.

”Well, the idea is fantastic but when it isn’t [a 100%] […] Only 20% wrong is enough, and it ruins everything. Even if it works 75% of the time, 80% of the time, it doesn’t matter” (Social worker, focus group 2).

Despite these potential harms, the importance of further developing these interventions was highlighted, given their high potential.

”I just want to say, that although we’ve underlined a lot of criticism, I really believe that we’ve got to continue to develop things like this. We shouldn’t give up, but learn from this” (Social worker, focus group 2).

## Discussion

### Main findings

This study identified two main themes describing clinicians’ experiences of using two novel app-based interventions in routine care, and several factors influencing implementation. The person-centred approach directed the treatment, emphasising that patients are expected to actively manage their own behavioural change. The interventions were helpful to clarify the active role of the patient within the treatment setting. Pertinently, clinician access to app-data was considered less important. Clinicians also thought that the apps supported behaviour change and considered the portals informative. Although the interventions were believed to have beneficial features for both patients and clinicians, there was need for further education and guidance on how to effectively use them. Moreover, there was a need for more easily accessible interventions (e.g., data integrated in already established technical systems), and specific worktime set aside to actively work with them. Technical issues, particularly with the breathalyser, were reported, undermining its reliability and reducing both patient and clinician motivation for continued use. Potential harms including data security of the apps and interpretation of BrAC-levels were also identified.

### Findings in context

In our study, the person-centred approach permeated the clinical work, and while access to patient data was considered a valuable add-on to the treatment, it was not always considered appropriate. Instead, some clinicians feared that their access to the data risked introducing a control system, threatening the person-centred approach. Historically, a control system and complete abstinence as treatment goal has been the norm, however not in conjunction with person-centred approaches and probably influenced by the substantial stigma surrounding alcohol dependence [38]. While clinicians in primary care previously have underscored that an app-based alcohol intervention would be better implemented without clinician support, the main reason was shortness of staff [22]. Relatedly, primary care patients have described clinician involvement and investment as essential for their engagement with app-based intervention [22], and alcohol-dependent patients have not considered app-based interventions suitable as standalone treatment [19, 39] and desired their integration in the treatment plan [23].

The fact that clinician use of the interventions was highly dependent on the patients’ app engagement further demonstrates the role of the patient taking the front seat. Asking patients what type of treatment that would best motivate them was described as a success measure in the forthcoming contact with patients, where apps could be offered as a complement for those who would be motivated by it, with or without clinician access. The importance of adapting treatment following each patient’s needs and technical skills, as well as prioritising trust-building with the patient has previously been underlined in studies among clinicians working with addiction in various settings, from elective care to inpatient treatment [40–42]. In our previous study on patient perceptions of using the app-based interventions, sharing data led to a higher accountability among some patients, but to negative effects for others, such as shame or embarrassment when failing to reach one’s consumption goal [19]. In turn, this resulted in reduced app use, or false use of the breathalyser (e.g., drinking after the last registration of the day) [19]. To minimize this risk, discussions on treatment expectations, initial goals, and reactions to failure ought to be discussed by the patient and the clinician at the onset of treatment [43].

Maintaining responsibility for the treatment process while supported by the clinician is a central principle of person-centred care and further represents an important factor for treatment adherence [44]. Accordingly, our study found that clinicians acknowledged that there was a higher patient-accountability among app-users, meaning that they took greater responsibility for the change process. Patients’ app use was further thought to better incorporate treatment into daily life. The benefits of using digital interventions to make patients more actively involved in their treatment has likewise been highlighted by clinicians working with psychiatric patients with co-occurring substance use disorders [40]. Moreover, clinicians in primary care have described that apart from offering alternatives to treatment, app-based interventions have potential in providing support between sessions [26].

Additionally, there was in the present study a belief that the digital registration of consumption was carried out more extensively than the traditional paper and pen calendar, and subsequently that patients that used the apps had increased consumption awareness, which facilitated discussion during treatment sessions. The constant accessibility of the smartphone has been identified as a valuable resource for patients [26]. Relatedly, patients have outlined that using apps as treatment complements offers benefits like easier registration, increased awareness and improved control [19]. Given that the frequency of tracking the amount consumed has been shown to mediate the effectiveness of apps targeted to reduced alcohol consumption [45], the notion that self-monitoring was more prominent using the digital compared to the paper calendar is particularly intriguing, and important to study further. Self-monitoring is a central component of behaviour change [46] and is an app-module that has been found to engage users to a high degree [47].

Contrasting the results with the Normalisation Process Theory (NPT) offers an opportunity for deeper understanding on key factors for successful implementation [28]. *Coherence* refers to the understanding, or sense-making, of the intervention [28, 48]. While clinicians described the interventions as beneficial both for themselves and their patients and stated the importance of using the interventions actively to enhance patient motivation, some had insufficient understanding of how they could utilise their full potential. This underscores the need to safeguard appropriate guidance and support. In line with these findings, the lack of a clear understanding has been described as a barrier for implementation [40], supporting the need for training and technical assistance when adopting mHealth interventions targeting substance use [42, 49].

*Cognitive participation* refers to clinicians’ commitment and engagement [28, 48]. Staff engagement is essential for sustained use of an implemented app-based intervention for alcohol dependence [49], and strategies to enhance engagement have been suggested in terms of integrating the intervention in weekly meetings, having peer-based training, and identifying motivated staff committed to working with the intervention [49]. Relatedly, the need for a dedicated person responsible for driving the intervention forward and setting up the procedures was emphasised in our study, as was the suggestion to become designated “app-therapists”, expressed as something that could aid commitment. Investing time and energy into the interventions was further hindered by lack of knowledge, and clinicians underscored the importance of valuing the interventions as beneficial to take it onboard.

As regards the operational work (*Collective action*) [28, 48], clinicians enacted on the interventions differently and incorporated them to various degrees in their routine work. When working with new technology within healthcare, ensuring staff’s digital skillset is crucial [20], and warrants adequate education and support. Critical resources needed to enable the interventions were in our study described in terms of additional worktime, easy access to the data, continuity in the work, and continuous (technical) support. While lack of technical skills was not explicitly discussed, clinicians were not prepared to solve the various technical issues encountered by patients, instead suggesting that dedicated personnel should handle these problems. As time constraints are common among clinicians, it is necessary to ensure feasible conditions regarding both workflow and time management. This is consistent with findings from prior research, where workflow, staffing and time have been identified as main constraints for implementing mHealth interventions for problematic substance use among clinicians in psychiatry and primary care [22, 25–27, 40]. The need for integrating technology-based interventions into routine systems (e.g., existing electronic records) has also been stressed [25, 49].

Regarding how clinicians valued the intervention as worthwhile (*Reflexive monitoring*) [28, 48], several critical factors were identified. Potential harms in terms of technical problems but also sub-optimal use of the apps, prompts, data security, and patient interpretations of their consumption, were discussed. In agreement with our results, previous research has acknowledged concerns about data security [22, 25, 26] and patient safety (e.g., reduced opportunities for patients to get validated in digital encounters) [27, 40]. Hence, these issues necessitate thoughtful consideration and identification of potential risks. Technical problems further jeopardised the reliability of the breathalyser. In a previous study investigating patient perceptions of using an app-coupled breathalyser within alcohol addiction aftercare, technical problems were also prominent [39]. These problems are probably a result of early development of this technology but still require attention and rigorous testing before implementation, as they may represent a major barrier to acceptance [19]. Still, clinicians described how the perceived usefulness of a consumption-tracking app or breathalyser led them to inform other patients about similar methods, apart from those already participating in the trial.

**Fig. 1:**
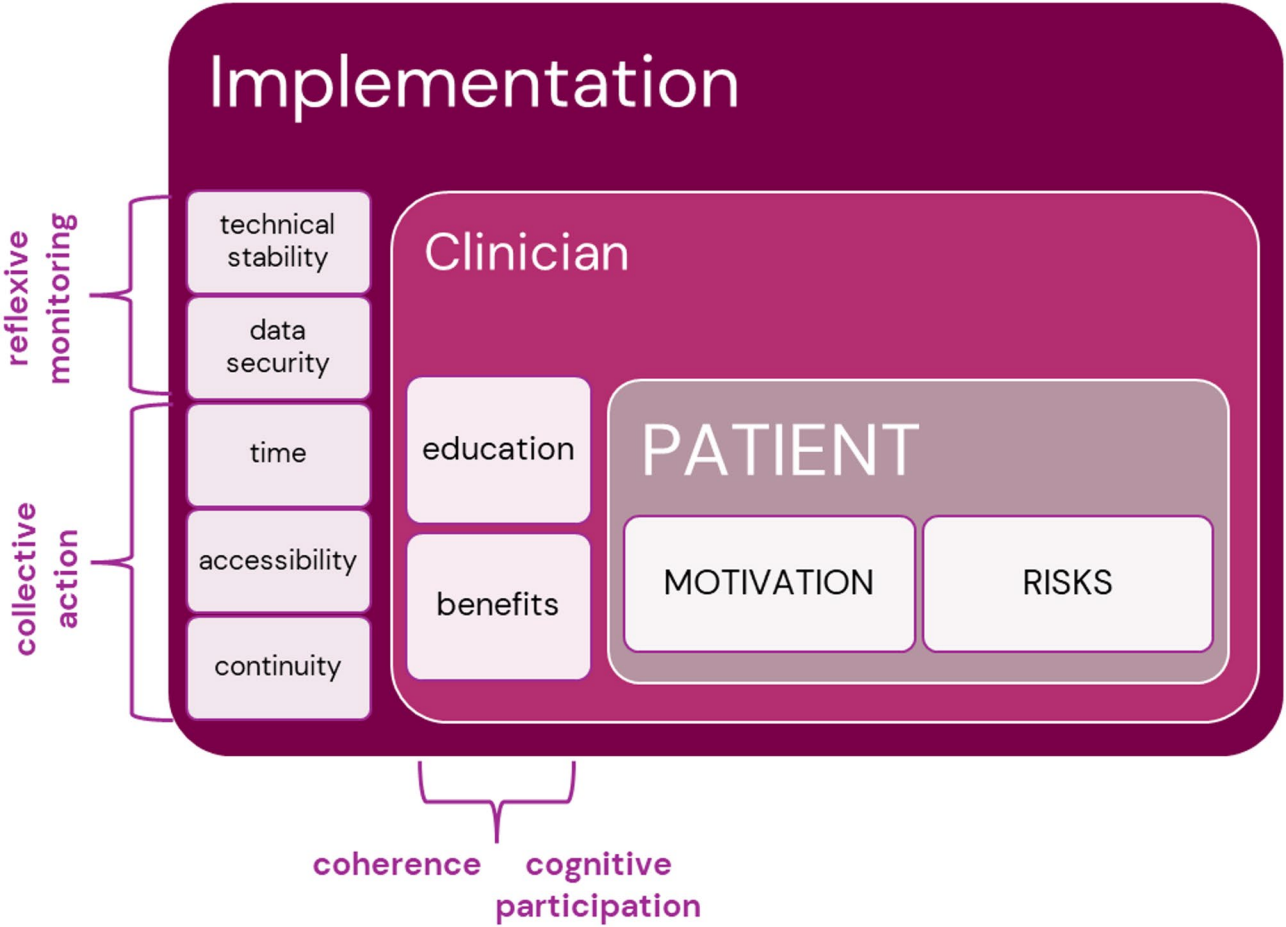
Overview of critical factors for successful implementation of smartphone-based alcohol interventions at an addiction clinic

To summarise, for successful implementation of mHealth into routine alcohol addiction treatment, various factors need to be considered. Although not part of the NPT, the patient is the central figure and driving force and chooses what would best support behaviour change. This is guided by the clinician making a careful investigation on who would benefit from using the interventions, to avoid potentially putting certain patients at risk for adverse events. Building further on this while incorporating the NPT constructs, the clinician needs to be well informed about the interventions, and value them as beneficial for routine treatment, which is a result of adequate information, education and support. Furthermore, there is need for continuity in the work (e.g., being a designated app-clinician), continuous support, additional time to review/discuss results, and enhanced accessibility (i.e., facilitated/integrated access to the web-based systems). Successful implementation further warrants technical stability and data security of the systems used. A summary of these findings is provided in Fig. 1.

### Strengths and limitations

Our sample was representative of the different professions at the clinic, but the final composition of groups was based on availability only. Hence, the first group comprised mostly females and nurses, and the second mostly men and psychologists, with various experience of working with the intervention. Nevertheless, all clinicians shared their experiences and expressed both concurring and opposing views in the discussions. To reduce the risk of general agreement among clinicians, the discussions could have been complemented with individual interviews [50]. However, this was not deemed possible with respect to the limited time resources of the clinicians. The discussions were rich with vivid descriptions and judged informative enough to cover the aim [51]. The clinicians were approached at a time when they recently had had study patients in treatment, which reduced the risk of limited recall of the intervention. Relatedly, the chosen method to use focus groups to discuss the intervention was further thought to aid recall. Importantly, the results of the RCT were not yet analysed at the time of data collection, which else could have influenced clinician views of the intervention.

Subjectivity could be regarded as a resource in qualitative research, where the authors’ own assumptions and experiences are reflected in the development of results [35, 52]. This, however, warrants a reflection on the active role of the authors, and how it directed research [35]. An attempt to provide a clear description is available in Supplement 3. The deductive use of NPT also directed research. Notably, and although extensively used, NPT has almost exclusively been evaluated in high-income settings [30].

This study contributes to a growing evidence base on clinician experiences of mHealth interventions and factors affecting their implementation in routine addiction care. However, it was conducted within the Swedish health care system which may affect the transferability of results. Relatedly, the clinic at which this study was conducted treats patients that fulfil the diagnostic criteria for alcohol dependence, without co-occurring drug dependence and with limited psychosocial problems. Therefore, this sample is not representative of all patients seeking addiction care, and results are not directly transferable to other contexts. Still, this sample may be reflective of the majority of alcohol-dependent individuals who have less extensive problem severity normally reluctant to seek care [53, 54]. Hence, the results of this study could potentially be transferable to primary care providers, that may encounter patients presenting similar problems.

## Conclusion

The results of this study indicate that app-based interventions have potential to be supportive complements to conventional treatment for both patients and clinicians. According to the clinicians, the app-based interventions made patients more aware of, and responsible for, their consumption, and supported treatment sessions. To effectively implement them into routine care, adequate education and support, continuity, extra worktime and integrated technical systems that facilitate easy access, as well as attention to patients’ individual motivations and possible risks are required. Furthermore, technical stability must be ensured.

## Supplementary Information

Below is the link to the electronic supplementary material.

Supplementary Material 1

## Data Availability

The data analysed in the current study are not publicly available due to confidentiality reasons but are available from the corresponding author upon reasonable request.

## Abbreviations

App: Smartphone application
BrAC: Breath alcohol concentration
FGD: Focus group discussions
mHealth: Mobile health
NPT: Normalisation Process Theory
RCT: Randomised controlled trial
TAU: Treatment as usual
WHO: World Health Organization
