# Supplementary material for: Clinicians’ perspectives on integrating smartphone application data into routine alcohol dependency treatment: factors influencing implementation
Source: Addict Sci Clin Pract. 2025 Aug 13;20:66. doi: 10.1186/s13722-025-00597-4 (PMC12344876; doi:10.1186/s13722-025-00597-4)
Supplement: Supplementary file 1 — Supplementary Material 1 [file 13722_2025_597_MOESM1_ESM.docx]

**Supplement 1 - Interview guide**

**Introduction**

Small talk, thank the clinicians for taking the time to participate, present study, and describe study aim, confidentiality, and use of data. It may be helpful to mention that there are no right or wrong answers. Describe our roles as moderator and secretary and invite for discussion. Mention the expected time frame and that it is ok to ask for a break during the interview. Also, clarify that participation is voluntary and that they have the right to cancel at any time. Ask if there are any initial questions before starting. Some group rules: to respect and not interrupt each other, turn off the mobile phone, and not share what is said during the discussion outside of this group.

**Main questions** *Start with Glasklart, continue with iBAC*

1. How did you use the portal during a usual treatment session?
   - What was its purpose, according to you?
   - Was there something that was difficult to understand?
   - Were there occasions where you did not use it?
2. What were the advantages of the portal?
   - Any factors/occasions that made it easier to use the portal?
3. What were the disadvantages of the portal?
   - What were the main barriers?
   - What was difficult?
   - Were there occasions when it was difficult using the portal?
4. What do you think of the various functions of the portal?
   - Glasklart: overview, risky drinking, alcohol type, cravings, mood
   - iBAC: scheduled and voluntary tests, breath alcohol concentration, diagram, picture
5. Was Glasklart/iBAC supportive?
   - In what way did you experience them as a support/not a support?
   - What is your experience – did the app make the patient drink less?
6. Would you consider using Glasklart/iBAC even after the end of the study?
   - Why/why not?
   - Do Glasklart/iBAC suit any particular patient? Why/why not?
7. According to you, how could technological aids, in this case apps, be used more generally in healthcare?
   - How could/should they be used?

**To finalize**

Is there something you would like to add? Anything important that we have forgotten to ask about or that has not been discussed that can give us a better understanding of how it has been to use Glasklart and iBAC (portals) as part of the treatment you give to patients? Thank you for your time and for sharing your valuable experiences.

**Follow-up questions**

Can you tell me more about that? In what way? Why? Have I understood you correctly if XXX? What do you mean with XXX? What did you think about XXX? You described XXX earlier, could you please elaborate a bit on that?

**Supplement 2 – Example of Matrix (Framework Analysis)**

| **Category** | **Codes** | **#1** | **#2** | **#3** | **#4** | **#5** | **#6** | **#7** | **#8** | **#9** | **#10** |
| --- | --- | --- | --- | --- | --- | --- | --- | --- | --- | --- | --- |
| **Balancing different patient needs** | **Individual specification, interactional workability, reconfiguration, adaptations** |  | Looking at the data together with the patient. More if used more regularly by the patient. Adaptations to treatment program thought necessary (p11). TAU protocol sufficient (p13). iBAC needs to be given to the "right" person (p34). | Most patients in the study had a goal to reduce consumption, which was new, if you usually work with abstinence (p8). Followed the protocols strictly without adjustments (p10). Patients needed more sessions (p13). It is important to show patients that breathalysers can be used in different ways (p33). | Adapting treatment after consumption goal, e.g., by adding craving scale for those with abstinence as treatment goal (p7). Everyone needs something relevant for them (p9). Appreciating the structure of TAU (p11), it was enough for patients (p13). You can ask patients what level of control they prefer as some might want clinicians to have control (p18). Would recommend patients to use breathalyser but not iBAC specifically (p32). | Giving advice to other patients (outside the study) that may benefit from Glasklart (p6). Added moderation strategies to the intervention (p11). Recommending all new patients to register consumption in Glasklart as an alternative (p19, 38). | Need to ask the patient what would best motivate them, and how (p26). Importance of connecting the intervention to internal and external motivation (p29). | Difficult to integrate in the treatment sessions, rather asking if used or not (p12). It gets patient-driven; for those who used the tools it was more discussed, but the majority wanted "normal" therapy sessions (p14). Have recommended other patients to use digital alcohol calendars (p15). Some patients want the extra control, want to show that they have zeros (p17). A patient-centered approach is already central at the clinic (p26). | If patients wanted to trick the system, it was a topic that could be discussed during treatment sessions (p3). How iBAC was used (motivation, relations) was what was important, not the technique (the HOW and not IF) (p4). | Did not sign into the portal frequently due to few patients (p9). If a patient was not interested in using Glasklart, the clinician tried to meet the patient in what motivated that patient (p9). The app is fortifying, beneficial for both patients and clinicians but treatment sessions were rather like they normally are (p13). What the patient wants must be a driving force (p19). You have to trust the patient (p22). Need to adapt after patients' needs, in a stepwise care model (p26). Different goals and motivation hindered a more structured treatment (p29). | Did not really incorporate the intervention into the treatment sessions, just asked about it, and since there already was a paper calendar, there was really no reason to use the tools. Did not want to burden the patient (p6). Patient engagement to the tools was the driving force (p14). Patients usually have different ambitions towards filling out alcohol calendars and some may like the digital form (p15-16). Importance of offering different options for the patients, and thus base treatment on patient needs and motivations (p32). |

**Supplement 3 – Reflexivity**

Writing this section was inspired by the following sources: (Trainor and Bundon 2021, Olmos-Vega, Stalmeijer et al. 2022, Braun and Clarke 2023).

I am the first author (JÖ), and I am a PhD-student with a background as a registered nurse, specialised in global public health. In the past six years, I have been a research nurse at the Clinic for Alcohol and Health (Riddargatan 1) in Stockholm. Although I have not myself had any patients in treatment, I know the staff at the clinic as we have been colleagues for many years, and I understand the context they work in. Relatedly, the idea for this study (examining clinicians’ views of the interventions) derived from my experience of being the study coordinator where I was the main contact person for patients, app-developers, but also for the clinicians, whose perspectives were important to further explore.

Keeping a reflexive diary was helpful to reflect on how my active role in data collection and analysis directed the research. Here below are some reflections that were noted during this process:

1. The questions in the interview guide mirrored those asked to patients in a previous study, including overall use, pros and cons, and perceived value. To remain neutral, questions were open-ended or had open-ended follow-up questions.
2. During interviews, I considered my insider-perspective as positive. The discussions were natural, the clinicians knew I had no conflicts of interests with the apps, and the topic was not sensitive. As both positive and negative experiences were brought forward, I felt that the clinicians did not feel reluctant to share their experiences, although it was a risk that our relationship as colleagues could affect the discussion. The clinicians had a lot of agreement amongst each other, but it was also evident that the discussion led some to think of the apps in a new light. As I had experience of being the study coordinator, I was well aware of the technical problems with mainly one of the apps that both patients and clinicians had experienced. Thus, it was a challenge to not just confirm and agree, but to also ask probing questions to remain a neutral and critical approach. I challenged myself to ask questions like “do you all agree with that” and so on, to make room for opposing views, and minimise the risk of directing the research based on my previous understanding of the apps.
3. By using a pre-defined set of codes, our approach was initially deductive. It felt good to have a framework to build upon, in contrast to starting from “zero”. Still, the coding process was not easy. As an active agent in this work, I applied the suitable codes based on my understanding of their definition. This iterative process was time consuming, and codes were revised a few times, also following discussions with the last author who provided valuable feedback. The use of the Normalisation Process Theory (NPT) directed the analysis in the sense that I started to think about the results in terms of its main constructs. The use of NPT was considered appropriate to identify factors that influence implementation, as NPT has been widely used and focuses on complex interventions. However, as we also had a more inductive aim to explore clinician views, sections that were not covered by NPT were added by applying open coding.
4. The inductive process of categorising codes further underlined my active role. As I feared losing context, I noted down clinician characteristics to keep them in mind. Furthermore, I wrote code definitions and key words from meaning units, that were read one by one, trying to detect similar patterns. A first set of categories were revised upon discussions with the last author. Writing clear descriptions of the category content was helpful, with contrasting views, also to generate overarching themes, that were discussed and derived together with the last author. Being aware of both the patients’ and clinicians’ experiences, however, probably influenced my interpretation. This is why the discussions, mainly with the last author, were extra valuable, also as we approached the data from different perspectives.

As the last author (AKD) and an associate professor of public health science, my background and role likely shaped the analysis of interviews with clinicians in dependency treatment. My position as a senior researcher, without clinical training or experience, may have influenced how I interpreted the clinicians’ narratives, and might have led to an emphasis on some certain themes, rather than others. Being removed from the direct practice of dependency treatment, my interpretations could reflect a broader perspective, which might diverge from the practical, day-to-day realities expressed by the clinicians. Recognizing this, I made a deliberate effort to remain attentive to their lived experiences, as expressed in the focus group interviews, and engaged in continuous discussions with the first author to ensure that diverse perspectives were considered throughout the analytical process.

The remaining authors included a doctor in epidemiology (AL), a professor in social alcohol and drug research (PW), and a senior professor of social medicine and medical doctor (SA). SA has long clinical experience working with treatment for alcohol dependence at the clinic but did not actively take part in data collection. Only the first author had access to the coding list, something the clinicians were aware of. Hence, the remaining team did not know who partook in the study, reducing the risk of unbalanced power. The diversity of the team was thought beneficial for the process, although all authors strive to improve addiction care.

**References**

Braun, V. and V. Clarke (2023). "Toward good practice in thematic analysis: Avoiding common problems and be(com)ing a knowing researcher." International Journal of Transgender Health **24**(1): 1-6.

Olmos-Vega, F. M., R. E. Stalmeijer, L. Varpio and R. Kahlke (2022). "A practical guide to reflexivity in qualitative research: AMEE Guide No. 149." Med Teach: 1-11.

Trainor, L. R. and A. Bundon (2021). "Developing the craft: reflexive accounts of doing reflexive thematic analysis." Qualitative Research in Sport, Exercise and Health **13**(5): 705-726.

**Supplement 4 – Examples of app and portal user interfaces**

**Glasklart drink-counting app interface (patient perspective):**


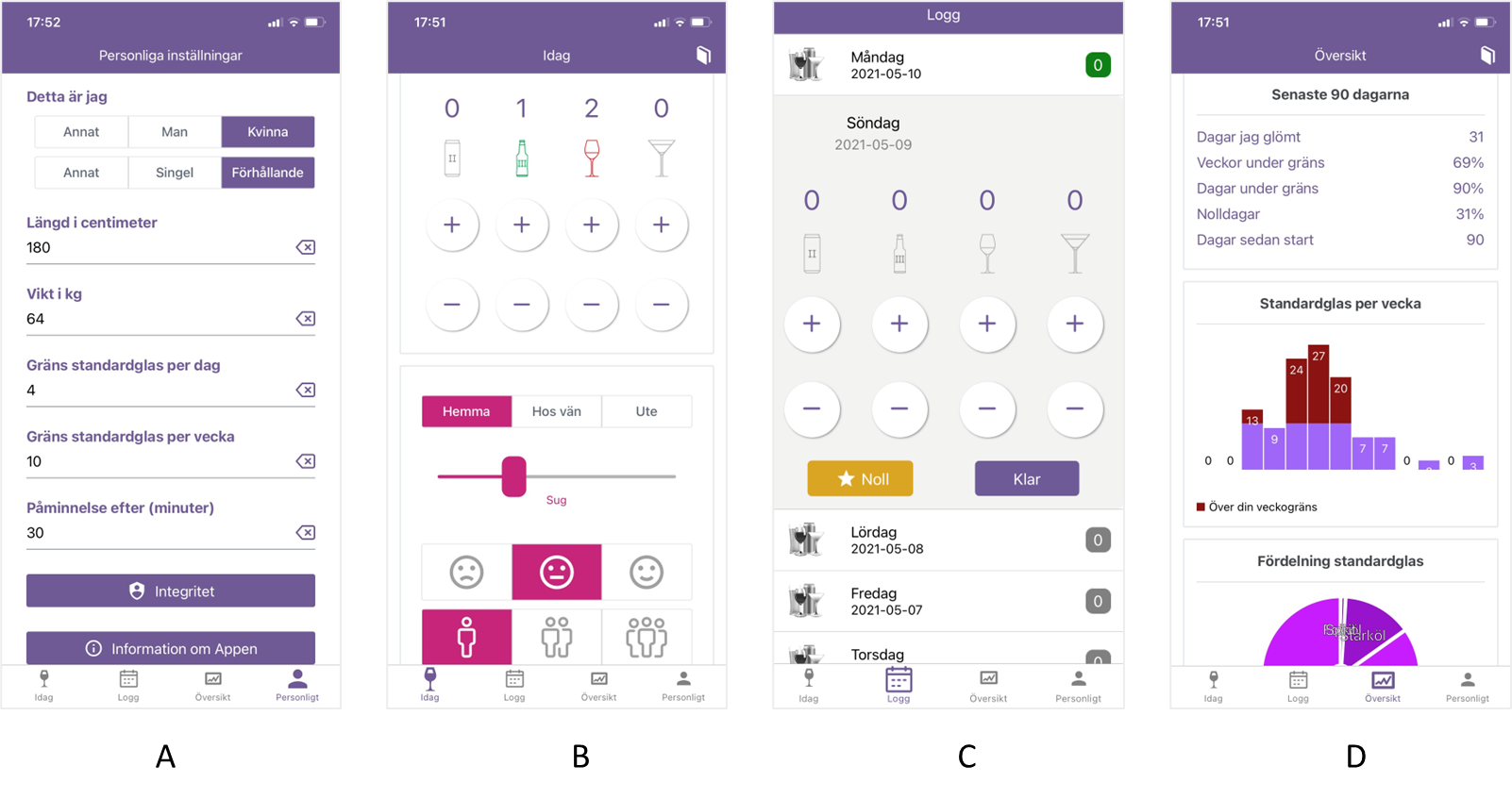


A) “Personal”: Personal details and goal setting (daily and weekly maximum of drinks).
B) “Today”: Daily/instant registration of consumption, location (home/at friend’s/out), craving (scale), mood (sad/neutral/happy), and company (alone/friend/group).
C) “Logg”: Registration of days with zero intake, consumption revision, and retrospective registrations.
D) “Översikt”: Overview of the consumption and additional parameters.

[LifeMesh - Glasklart](https://lifemesh.se/)

**Example of Glasklart portal (below) viewed from the clinician’s perspective:**


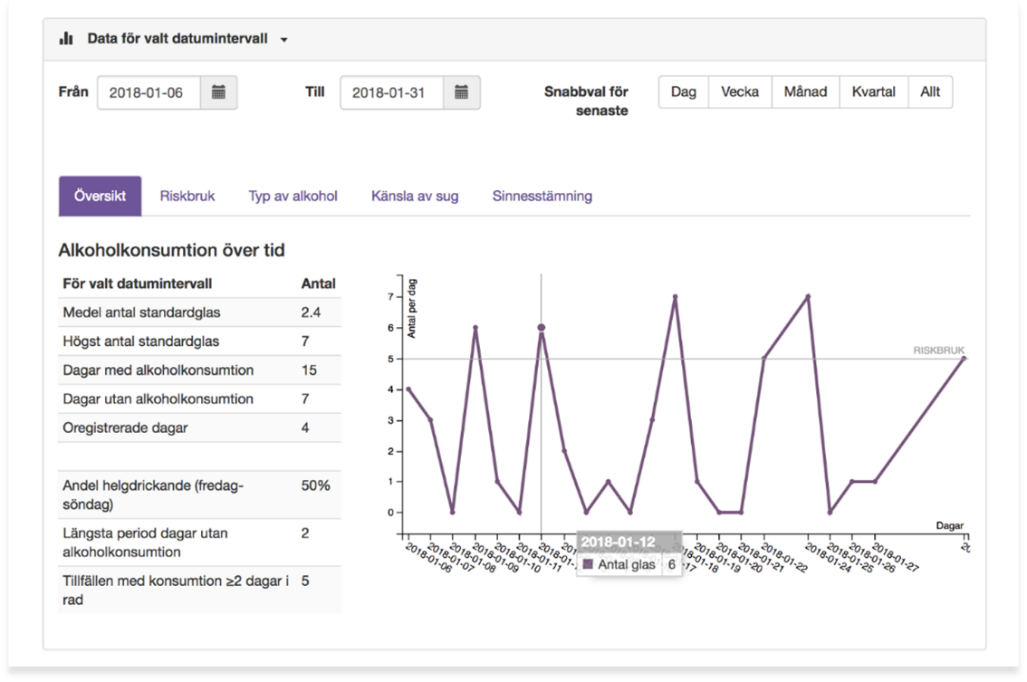


Overview, including registered drinks per day during a chosen time interval, mean and maximum number of drinks, number of days with/without consumption, and proportion of weekend consumption.


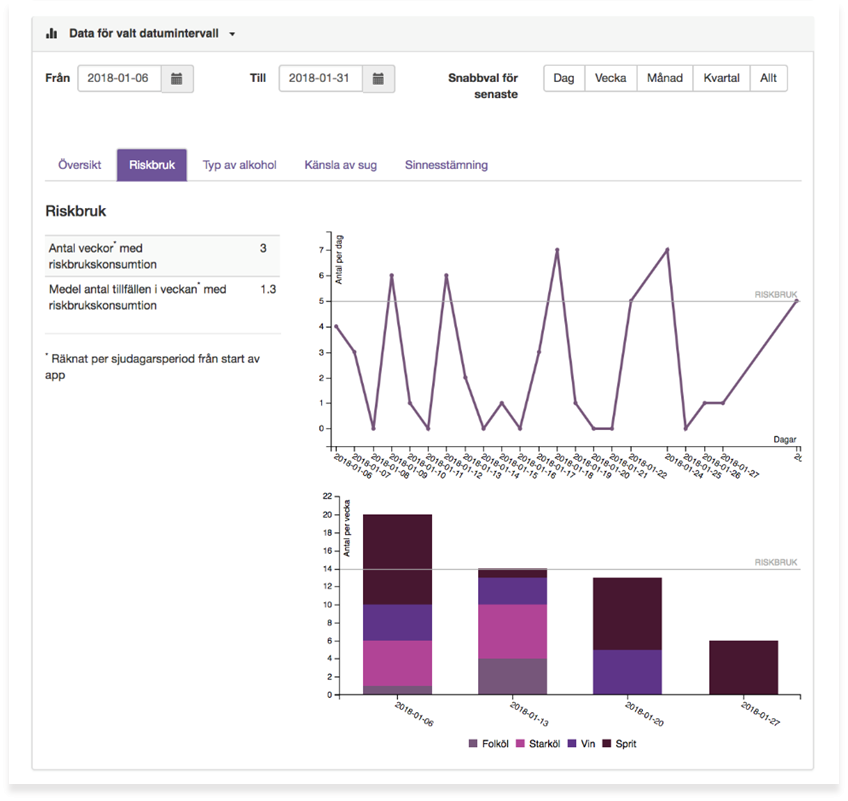


Hazardous drinking, including weeks with hazardous drinking, and mean days per week with hazardous consumption.

**Interface of iBAC Pro app and coupled breathalyser (patient perspective):**


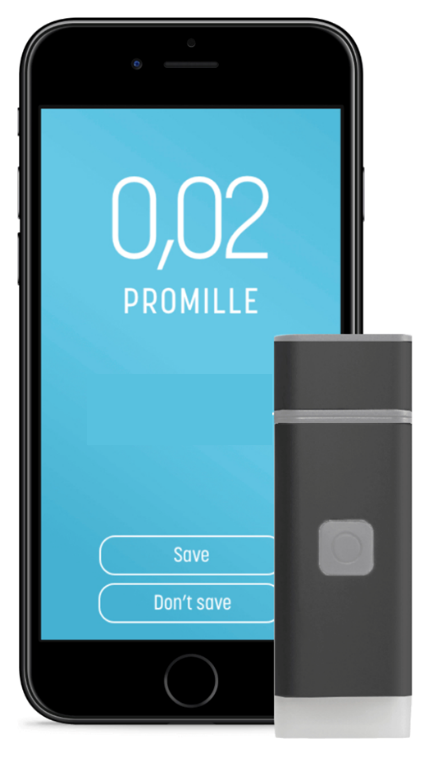


Breathalyser connected to smartphone app by Bluetooth. Picture from: <https://myibac.se/ibac/>


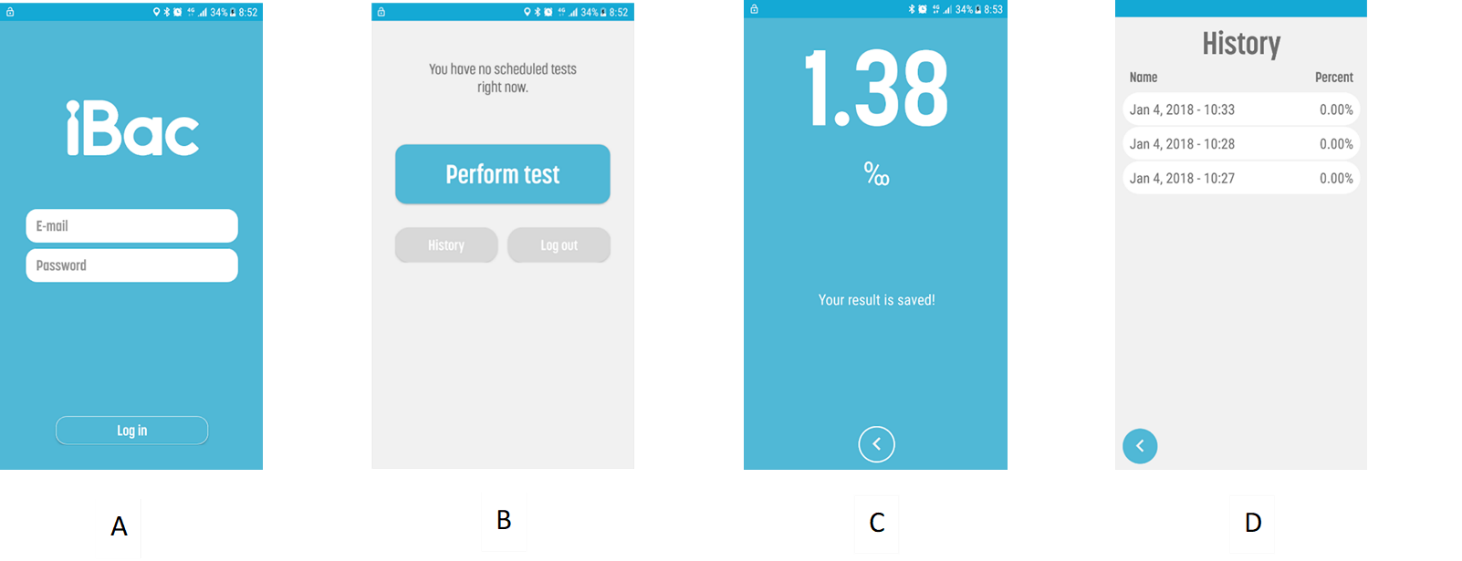


A) Sign-in start page.
B) Performing a test.
C) Instant test result available in the app.
D) History of previous tests.

**Example of iBAC Pro portal (below) viewed from the clinician’s perspective:**


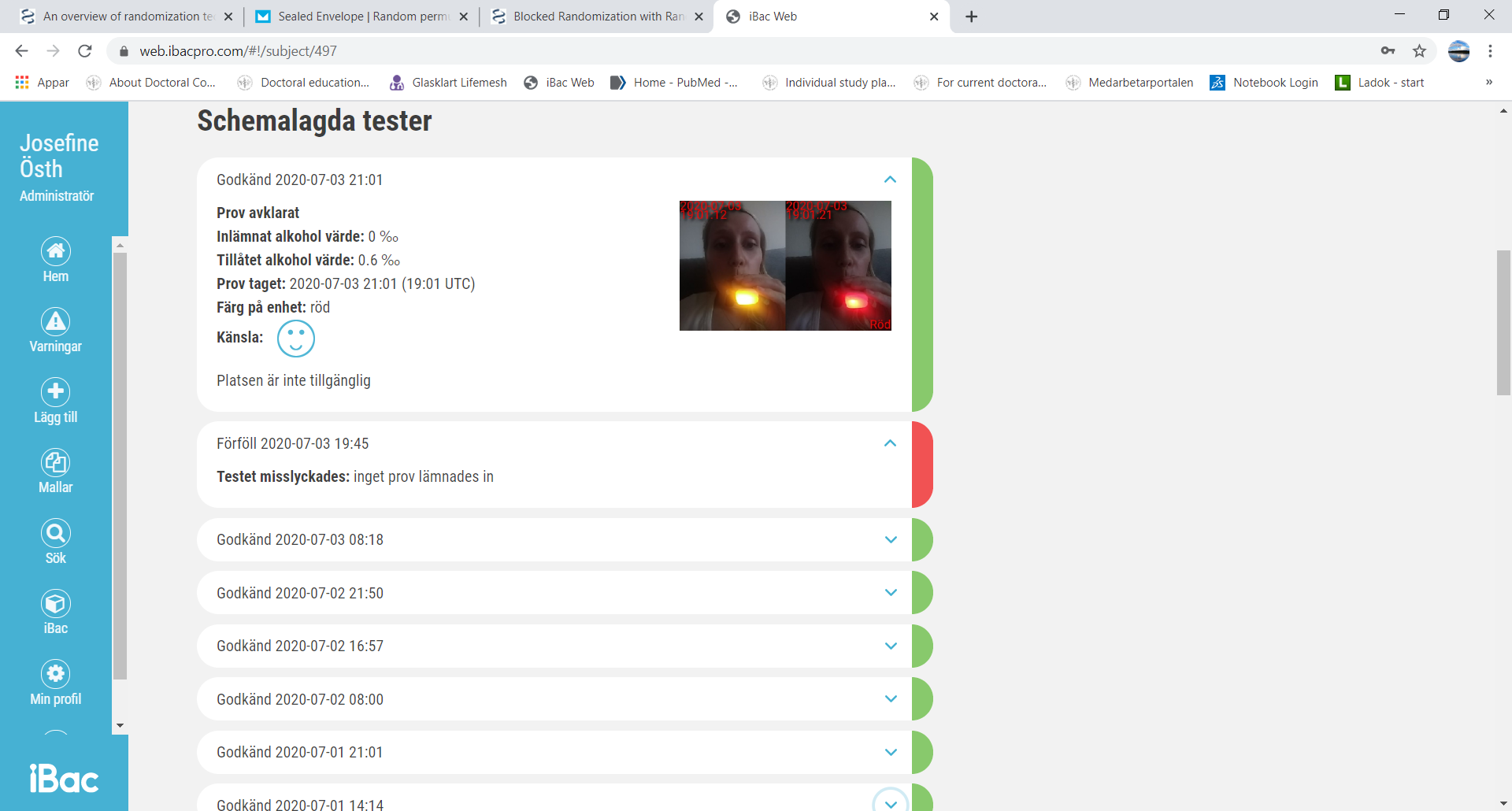


Scheduled and spontaneous breath alcohol concentration tests. Green=performed test with a result under 0.06 g%. Red=non-performed test, or test result >0.06 g%. (NB: this output is from when the main author tested the device, study participants were instructed to re-direct their phone camera if they did not want their picture taken).


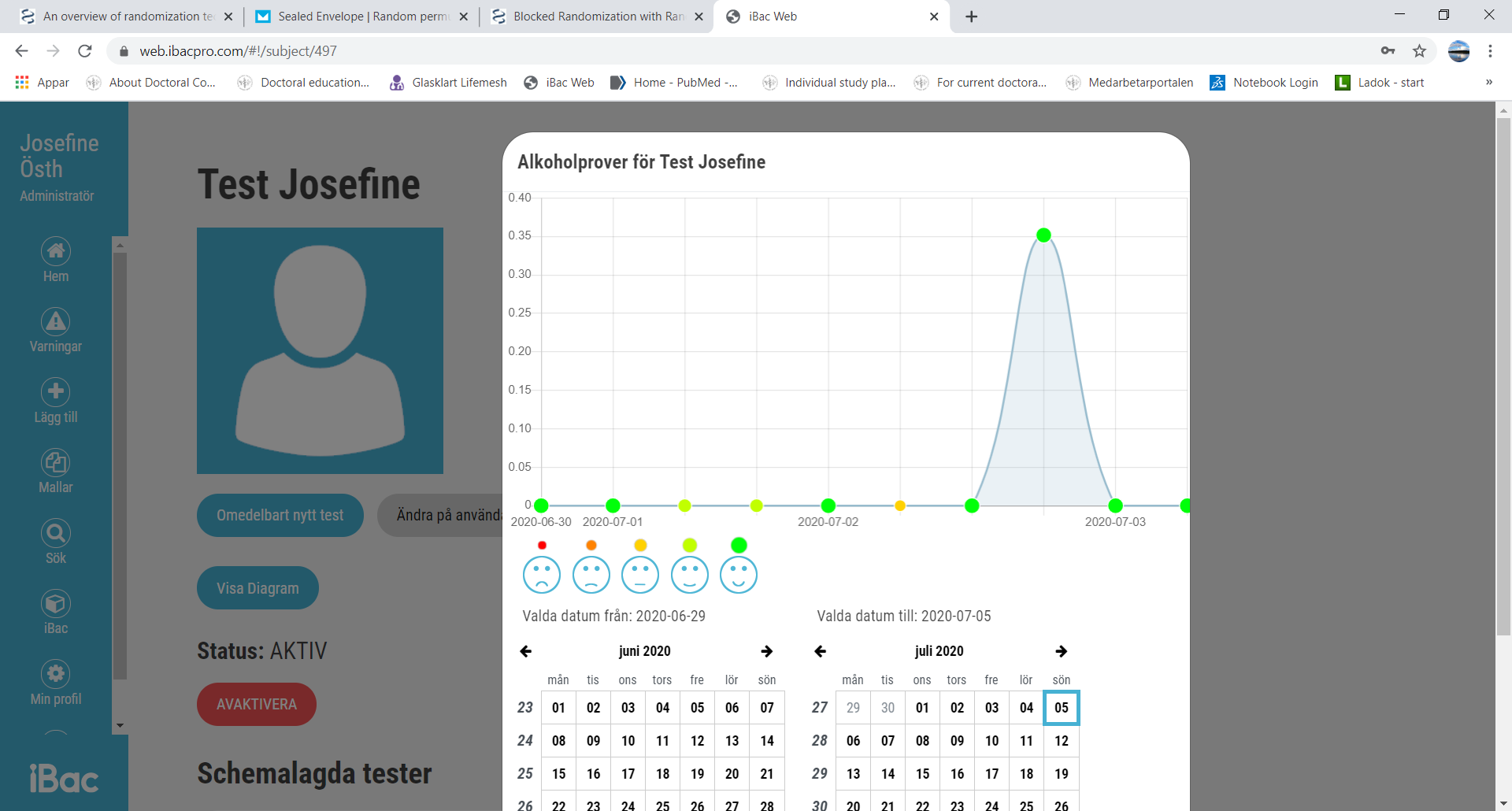


Diagram of daily peak breath alcohol concentration tests, and related mood (colour indicated) during a chosen time interval.
